# Superior fixation and less periprosthetic stress-shielding of tibial components with a finned stem versus an I-beam block stem: a randomized RSA and DXA study with minimum 5 years’ follow-up

**DOI:** 10.1080/17453674.2019.1566510

**Published:** 2019-01-23

**Authors:** Maiken Stilling, Inger Mechlenburg, Claus Fink Jepsen, Lone Rømer, Ole Rahbek, Kjeld Søballe, Frank Madsen

**Affiliations:** a Orthopaedic Research Unit, Aarhus University Hospital;;; b Centre of Research in Rehabilitation (CORIR), Department of Clinical Medicine, Aarhus University Hospital and Aarhus University;; c Department of Orthopaedic Surgery, Aarhus University Hospital;; d Department of Radiology, Aarhus University Hospital;; e Department of Clinical Medicine, Aarhus University, Denmark

## Abstract

Background and purpose — The stem on the tibial component of total knee arthroplasty provides mechanical resistance to lift-off, shear forces, and torque. We compared tibial components with finned stems (FS) and I-beam block stems (IS) to assess differences in implant migration.

Patients and methods — In a patient-blinded RCT, 54 patients/knees (15 men) with knee osteoarthritis at a mean age of 77 years (70–90) were randomly allocated to receive tibial components with either a FS (n = 27) or an IS (n = 27). Through 5 to 7 years’ follow-up, implant migration was measured with RSA, periprosthetic bone mineral density (BMD) was measured with DXA, and surgeons reported American Knee Society Score (AKSS).

Results — At minimum 5 years’ follow-up, maximum total point motion (MTPM) was higher (p = 0.04) for IS (1.48 mm, 95% CI 0.81–2.16) than for FS (0.85 mm, CI 0.38–1.32) tibial components. Likewise, total rotation (TR) was higher (p = 0.03) for IS (1.51˚, CI 0.78–2.24) than for FS (0.81˚, CI 0.36–1.27). Tibial components with IS externally rotated 0.50° (CI –0.06 to 1.06) while FS internally rotated 0.09° (CI –0.20 to 0.38) (p = 0.03). Periprosthetic bone stress-shielding was higher (p < 0.01) up to 2 years’ follow-up for IS compared with FS in the regions medial to the stem (–13% vs. –2%) and posterior to the stem (–13% vs. –2%). Below the stem bone loss was also higher (p = 0.01) for IS compared with FS (–6% vs. +1%) up to 1-year follow-up. Knee score improved similarly in both groups up to 5 years’ follow-up.

Interpretation — Periprosthetic bone stress-shielding medial and posterior to the stem until 2 years, and tibial component migration at 5 years, was less for a finned compared with an I-shaped block stem design.

Fixation of the tibial component in total knee arthroplasty (TKA) can be augmented with different stem designs. The classic stem shape is either a central block or cylinder with or without fins (Hernandez-Vaquero et al. 2008), which provides mechanical resistance to lift-off, shear forces, and torque during knee kinematics (Grupp et al. 2017). The smaller the tibial stem, the less bone is lost at implantation, and potentially preserved for later revisions (Molt and Toksvig-Larsen 2015). A finned stem design provides a greater mechanical resistance to torque than a block stem (Hernandez-Vaquero et al. 2008). A further advantage of a finned stem compared with a cylindrical or a block stem is that less bone volume is removed and the periprosthetic bone is exposed to less stress (Hernandez-Vaquero et al. 2008, Molt and Toksvig-Larsen 2015). Therefore, improved fixation and survival could be expected with a finned stem design. With cemented TKA fixation primary stability of the components is secured initially and longer term fixation relies on the quality of the fixation interfaces, including cement penetration and bone quality (Vertullo and Davey 2001, Andersen et al. 2017). Early migration of the tibial component measured with radiostereometric analysis (RSA) is predictive for an increased risk for subsequent loosening at a later stage (Ryd et al. 1995, Pijls et al. 2012).

We compared tibial components with an I-beam stem (IS) and a finned stem (FS) at 2 and minimum 5 years’ follow-up in a randomized trial evaluating RSA-measured implant migration and polyethylene wear, changes in periprosthetic bone mineral density (BMD), and differences in clinical outcome measured by the American Knee Society Score (AKSS).

## Patients and methods

**Figure 1.**
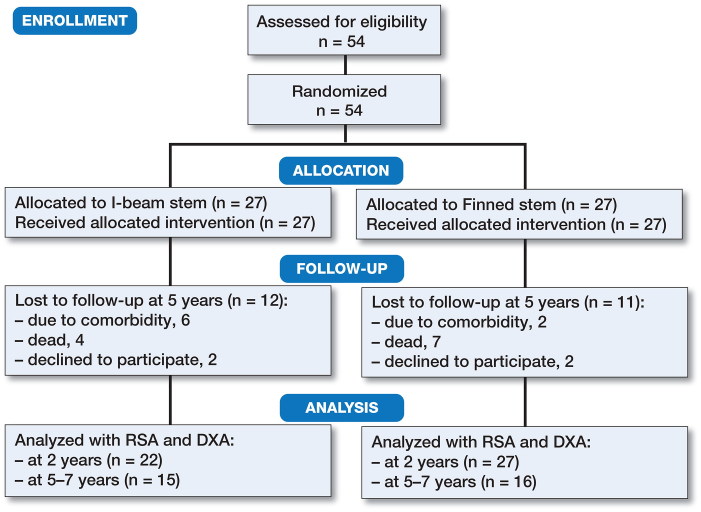
CONSORT flow diagram showing the inclusion/exclusion process and follow-up until minimum 5 years

Between January 2005 and December 2007, 54 patients with primary osteoarthritis of the knee were assessed for eligibility in this single-center patient-blinded randomized controlled clinical trial. Randomization in blocks of 6 patients (3 IS and 3 FS) was done by drawing labels from a box, and the labels were then concealed in 54 consecutively numbered closed envelopes. All eligible patients gave their informed consent to participation to the surgeon and received allocation intervention at Aarhus University Hospital, Denmark (Figure 1, Table 1).

**Table 1.** Demographics, surgical and clinical data at baseline

Factor	I-beam stem (n = 27)	Finned stem (n = 27)
Men/women	8/19	7/20
Operated side (right/left)	12/15	12/15
Age at surgery (mean, range)	77 (70–90)	77 (70–85)
BMI at surgery (mean, range)	28 (20–37)	29 (21–37)
Number of surgeons	4	4
Implant size (range)	71–83	69–83
Polyethylene thickness (mm)	10 (8–12)	10 (8–12)
Surgery time (min)	63 (45–85)	48 (48–90)
AKSS (max 100) (mean, range)		
Knee Score	34 (13–70)	36 (10–62)
Function Score)	45 (0–70)	54 (15–90)

The inclusion criteria were primary osteoarthritis of the knee, age above 70 years, informed consent, and only one knee operated. The exclusion criteria were severe neuromuscular or vascular disease of the lower extremities, insufficient bone quality for a TKA, known osteoporosis, previous proximal tibial osteotomy, or other major knee surgery.

### Calculation of sample size

The primary end point was 2 years’ follow-up, and with minimal relevant difference of 0.5 mm maximum total point motion (MTPM) (power 90%, alpha 0.05, standard deviation 0.6 mm) (Ryd et al. 1995) this study was powered for 22 patients per group. To compensate for eventual dropouts, 27 patients per group were included (Figure 1).

### Implants

**Figure 2.**
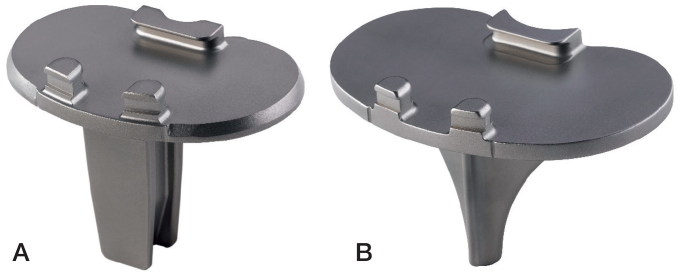
The Maxim Total Knee (Zimmer Biomet, Warsaw, Indiana) with cobalt-chromium Tibial Tray Interlok components with (A) an I-beam stem and (B) a finned stem.

Maxim Total Knee (Zimmer Biomet, Warsaw, IN, USA) cruciate-retaining components were used. The cobalt-chromium modular Tibial Tray Interlok had either an I-beam block stem or a finned stem (Figure 2). Both stem types were 4 cm long and fixed to the tibial baseplate (non-modular). Both components were fixed in the bone by vacuum-mixed Palacos R bone cement (Heraeus Medical GmbH, Wehrheim, Germany) applied under the baseplate while the stem was fixed press-fit (without cement) in the proximal tibia. The femoral component Maxim cobalt-chromium (Biomet Inc, Warsaw, IN, USA) and the patella resurfacing polyethylene was fixed by Palacos R bone cement. The polyethylene insert was a modular component of gamma sterilized Arcom (Zimmer Biomet, Warsaw, IN, USA) ultra-high molecular weight polyethylene fixed with similar locking splits in IS and FS components (Figure 2).

### Surgery

All patients were operated in a theatre with laminar airflow by 4 experienced knee surgeons. A tourniquet was applied and an anterior midline incision was used. The posterior cruciate ligament was retained in all cases. In both groups the proximal tibia was cut using the same extra-medullary guide, aiming for a perpendicular cut in the frontal plane and a posterior slope of 3°. The cut surfaces of the patella and femur were cleaned by high-pressure lavage before cementation. 5–6 tantalum beads (1 mm) (Wennbergs Finmek AB, Gunnilse, Sweden) were inserted in the proximal tibia intraoperatively. All patients received a draining tube in the joint for approximately 24 hours. All patients were treated prophylactically with a preoperative single dose i.v. 2 g dicloxacillin and all received prophylactic thrombotic medication with 1 daily dose subcutaneous 2.5 mg fondaparinux for 5 to 7 days. The patients were mobilized on the first postoperative day and allowed weight-bearing as tolerated, but with the assistance of 2 crutches for the first 6 weeks. The in-hospital stay varied between 4 and 6 days.

### Radiosteometric analysis (RSA)

Stereoradiographs were obtained within the first 2–3 days after surgery (reference examination). Subsequent examinations were obtained at 6 weeks, 3 months, at 1 and 2 years, and cross-sectionally at 5 to 7 years with the patient supine and the operated knee aligned parallel to the calibration box (y-axis) in a foam positioner. The position and orientation of the global coordinate system in the reference examination defined the direction of implant migration in the follow-up examinations. At 5 years standing stereoradiographs (30° knee flexion) were also obtained. We used a fully digitized standard RSA setup (FCR Profect CS; Fujifilm, Vedbaek, Denmark) with 2 synchronized ceiling-fixed roentgen tubes (Arco-Ceil/Medira; Santax Medico, Aarhus, Denmark) angled 40° on each other and an unfocused uniplanar carbon calibration box (Box 24, Medis Specials, Leiden, the Netherlands). Analysis was performed with Model Based RSA vs. 3.21 (RSAcore, Leiden, The Netherlands) using CAD implant models (Kaptein et al. 2007). The signed migrations described the general migration of the implants, and the maximum total point motion (MTPM) described the vector of the point of the implant model that migrated the most. Implants were classified as stable or continuously migrating based on the MTPM, as described by Ryd et al. (1995). Further, we calculated the total rotation (absolute implant rotation) using the Pythagorean theorem (TR = √X^2^+Y^2^+Z^2^). Polyethylene wear was measured as loss of joint space width by calculating the migration difference on the y-axis between the femoral and tibial model components at 5 years (standing) stereoradiographs with postoperative (supine) stereoradiographs as baseline. The condition number of the bone marker model was mean 43 (SD 22) and the rigid body error was mean 0.17 (SD 0.1). Guidelines for maximum CN (< 150) and ME (0.35) were used as upper limits (Valstar et al. 2005), and no RSA analyses were excluded by these criteria.

The repeatability of the RSA measurements was calculated based on double stereoradiographic examinations of 29 patients (15 IS and 14 FS) at the last follow-up (Valstar et al. 2005). The postoperative stereoradiograph was used as the reference in migration analysis of the double examinations (Table 2, see Supplementary data).

### Dual-energy X-ray absorptiometry (DXA)

**Figure 3.**
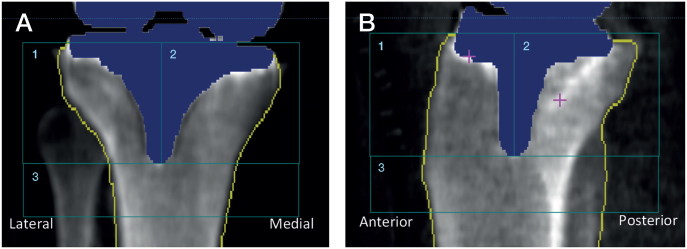
DXA scans showing the implant detection of the finned stemmed implant (blue), bone borders (yellow line), and 3 regions of interest (ROI) around the stem. A. Anterior/posterior view. B. Lateral view.

DXA scans of the periprosthetic bone were performed within the first postoperative week (baseline), and at 1 and 2 years, and cross-sectionally at 5 to 7 years using a Lunar Prodigy DXA Scanner (GE Healthcare, Waukesha, WI, USA). The patients were positioned supine with the leg in a foam frame to keep the knee semi-flexed by approximately 25° and the lower leg in neutral rotation (Stilling et al. 2010). Rice was applied around the knee as tissue-equivalent material and scans were performed with the “spine” mode. Analysis was performed in 3 regions of interest (ROI) (Figure 3) with a precision range from 1.8% to 3.7% for the anteroposterior scans, and from 3.4% to 6.2% for the lateral scans (Stilling et al. 2010).

### Clinical follow-up

The patients were seen for clinical examination preoperatively, and at 1 and 2 years, and cross-sectionally at 5 to 7 years postoperatively. Clinical data collection was conducted unblinded by the 4 surgeons. The AKSS was used to quantify the functional result, patient satisfaction, and pain (Insall et al. 1989).

### Statistics

All continuous variables were tested for normality (Shapiro Wilk test). The groups were then compared by a 2-sample t-test or a 2-sample t-test with unequal variances as appropriate. Non-normal data were tested by a 2-sample Wilcoxon rank-sum (Mann–Whitney) test. Spearman’s rho was used to test correlations between implant migration, bone-density changes, patient factors, and clinical outcome. The statistical analyses were performed using the STATA 14.0 (StataCorp, College Station, TX, USA) software package. The significance level was set at 0.05.

### Ethics, funding, and potential conflicts of interest

The Central Denmark Region Committee on Biomedical Research Ethics approved the protocol (Journal Number: M-20030239), which was registered with ClinicalTrials.gov (NCT00175136), and performed in compliance with the Helsinki Declaration. Informed consent was obtained from all participants. Zimmer Biomet, the Danish Rheumatism Association, and the A.P. Møller Foundation unconditionally sponsored the study. The authors declare no conflicts of interest.

## Results

### RSA

**Figure 4.**
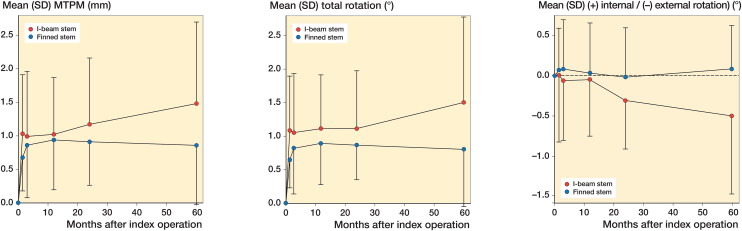
Line plot summarizing the MTPM, total rotation (TR), and rotation about the y-axis (internal–external rotation) of the tibial components in the 2 stem groups at 6 weeks, 3 months, 1 year, 2 years, and minimum 5 years. Between 2 and minimum 5 years’ follow-up there was a statistically significant difference in all 3 migration parameters showing higher migration in the tibial components with I-beam stem (red) compared with finned stem (blue). The dots mark the means and the error bars are standard deviations.

1, 2, and minimum 5 years’ RSA data are shown in Figure 4 and Table 3. There was similar implant migration between groups until 2 years’ follow-up. On group level, there was no measurable migration between 1 and 2 years for IS (p = 0.3) or for FS (p = 0.7). Between 1 and 2 years 21 tibial components (12/22 IS and 9/27 FS) were classified with continuous migration (MTPM > 0.2mm) and 28 were stable (10/22 IS and 18/27 FS) (p = 0.1) (Ryd et al. 1995). At 2 years, 5/21 of IS and 5/27 of FS had rotation of more than 0.7° about the y-axis (p = 0.7) (Gudnason et al. 2017), and at 5 years this was 4/14 in both groups. Between 2 and minimum 5 years there was no measurable migration at group level (p > 0.09); however, 19 tibial components (12/15 IS and 7/16 FS) had MTPM > 0.2 mm and 12 were stable (3/15 IS and 9/16 FS) (p = 0.04).

**Table 3.** Signed migrations of the tibial components at 1, 2, and minimum 5 years’ follow-up. Values are mean (95% CI)

	I-beam stem	Finned stem	p-value **^c^**
x–translation (+medial/–lateral):			
1 year	0.05 (–0.12 to 0.22)	–0.02 (–0.12 to 0.07)	0.5
2 years	0.04 (–0.18 to 0.27)	–0.03 (–0.12 to 0.07)	0.5
5 years	–0.20 (–0.50 to 0.09)	0.02 (–0.06 to 0.10)	0.5
y–translation (+lift-off/–subsidence):			
1 year	0.09 (0.01 to 0.17)	0.09 (0.01 to 0.17)	1.0
2 years	0.12 (0.06 to 0.18)	0.10 (0.01 to 0.19)	0.6
5 years	0.15 (0.05 to 0.25)	0.08 (–0.05 to 0.21)	0.3
z–translation (+anterior/–posterior):			
1 year	0.07 (–0.23 to 0.37)	–0.22 (–0.45 to 0.01)	0.5
2 years	–0.07 (–0.43 to 0.29)	–0.16 (–0.36 to 0.04)	0.4
5 years	–0.39 (–0.89 to 0.10)	–0.07 (–0.39 to 0.25)	0.5
MTPM **^a^**			
1 year	1.02 (0.65 to 1.40)	0.94 (0.65 to 1.24)	0.8
2 years	1.17 (0.72 to 1.61)	0.91 (0.65 to 1.17)	0.8
5 years	1.48 (0.81 to 2.16)	0.85 (0.38 to 1.32)	0.04
x–rotation (+anterior tilt/–posterior tilt):			
1 year	0.15 (–0.34 to 0.64)	–0.33 (–0.64 to –0.02)	0.2
2 years	–0.05 (–0.52 to 0.41)	–0.22 (–0.51 to 0.07)	0.4
5 years	–0.52 (–1.33 to 0.29)	–0.12 (–0.66 to 0.42)	0.9
y–rotation (+internal rotation/–external rotation):			
1 year	–0.05 (–0.37 to 0.26)	0.03 (–0.19 to 0.19)	0.7
2 years	–0.31 (–0.59 to –0.04)	–0.02 (–0.27 to 0.22)	0.2
5 years	–0.50 (–1.06 to 0.06)	0.09 (–0.20 to 0.38)	0.03
z–rotation (+varus/–valgus):			
1 year	0.02 (–0.21 to 0.26)	0.04 (–0.10 to 0.17)	1.0
2 years	0.00 (–0.32 to 0.32)	0.03 (–0.10 to 0.16)	0.7
5 years	0.39 (0.01 to 0.76)	–0.01 (–0.14 to 0.13)	0.1
TR **^b^**			
1 year	1.12 (0.75 to 1.48)	0.90 (0.66 to 1.14)	0.5
2 years	1.12 (0.73 to 1.50)	0.88 (0.67 to 1.08)	0.5
5 years	1.51 (0.78 to 2.24)	0.81 (0.36 to 1.27)	0.04

a MTMP: maximum total point motion.

b The total rotation (TR) was calculated using the 3D Pythagorean theorem.

c Difference between groups (two-sample Wilcoxon rank-sum (Mann–Whitney) test).

We found no clinically relevant or statistically significant correlations between implant migration (MTPM) and sex (p = 0.9) and BMI (p = 0.6). There was a correlation between MTPM at minimum 5 years’ follow-up and periprosthetic bone loss percentage medially (rho –0.38; p = 0.03) under the tibial component.

There was 68% more (p = 0.04) polyethylene wear of tibial components with IS (1.05 mm, CI 0.64–1.46) compared with FS (0.48 mm, CI 0.06–0.89). The wear-rate (mean and (standard deviation)) of polyethylene inserts in tibial components with IS and FS was 0.21 (0.15) mm/year and 0.10 (0.15) mm/year, respectively.

### DXA

IS had more periprosthetic stress-shielding (bone loss) (p < 0.01) up to 2 years’ follow-up in the regions medial to the stem (–13% vs. –2%) and posterior to the stem (–13% vs. –2%) compared with FS (Table 4, see Supplementary data). Below the stem, bone loss was also more pronounced (p = 0.01) for IS compared with FS (–6% vs. +1%) up to 1-year follow-up. Between 2 and minimum 5 years the bone loss medially, laterally, and below the stem decreased markedly for FS (p < 0.002) whereas there was no statistically significant difference for IS (p > 0.2).

### Clinical results

There were no intraoperative complications. 1 patient (IS) had a postoperative superficial infection that resolved after antibiotic treatment, and 1 patient (FS) had an early deep infection that was treated with reoperation including lavage and exchange of the polyethylene liner plus 6 weeks of antibiotic treatment, and healed without further complications. There were no revisions within 5 years’ follow-up. Clinical results were similar between the 2 groups of patients at minimum 5 years of follow-up (Table 5, see Supplementary data). Knee score and function score improved until 1 year but not thereafter. 27 patients had an extension deficit at baseline; all except 2 patients regained full extension (5 and 10 degrees extension deficit).

## Discussion

This is the first study to compare tibial components with different stem designs in a randomized study. The key findings were that tibial components with finned stem in comparison with I-beam block stem had less periprosthetic bone stress-shielding up to 2 years, and maintained better fixation and had less polyethylene wear at 5 years.

### Fixation and survival

Although cemented components achieve stable fixation during surgery, they normally present with a pattern of some initial migration until 3 months and thereafter stabilization until small individual component migration become indicative of later aseptic loosening (Ryd et al. 1995). Classically, cemented metal-backed tibial components migrate via tilting subsidence with lift-off and loosening (Gudnason et al. 2017).

The summed migration measures (MTPM and TR, Figure 4) in our study indicated more migration in the IS group already at 6 weeks, but there was no progressive migration at group level until after 2 years’ follow-up. At 1-year follow-up, the mean MTPM in both groups was slightly higher than the reported mean of 0.7 mm for the AGC TKA in the meta-analysis study of Pijls et al. (2012) which placed the implants in our study in the “at risk” group (0.45–1.6 mm MTPM at 1 year), with a higher than 5% revision risk at 10 years. Ryd et al. (1995) reported MTPM around 1 mm at 5 years and 1.2 mm at 10 years in non-revised cemented tibial components. Between 2 and minimum 5 years, the IS group had mean MTPM of 1.48 mm in 12 of 15 patients displaying continuous tibial component migration, whereas the FS group had mean MTPM of 0.85 mm in 7 of 16 patients displaying continuous tibial component migration. We did not have any revisions until the cross-sectional 5- to 7-year follow-up in this study apart from a liner exchange during 1 infection reoperation. The Finnish Knee Arthroplasty Registry have shown a 94% 10-year survival rate in osteoarthritic knees operated with the AGC TKA (Biomet), a cemented tibial platform with an I-shaped block stem, which is the predecessor of the Maxim TKA (Biomet) of this study (Himanen et al. 2005). In a large case series, Faris et al. (2015) reported aseptic 10-year survival of 98.7% for the AGC TKA system (n = 5,972) and 98.5% survival rate for the Vanguard TKA system (n = 1,209). The Vanguard TKA system has a finned stem and is the successor of the Maxim TKA (Biomet) with a finned stem used in this study. The arthroplasty registries do not report the stem design of the tibial components, and it is unclear how many tibial components have a fixed or modular IS or FS stem besides the AGC TKA system, the Maxim TKA system, and the Vanguard system. Further, it is unclear whether survival of IS and FS in these TKA brands are different.

At minimum 5 years we found higher total rotation for IS compared with FS, which showed as a difference in signed rotation about the y-axis, as 0.5° external rotation for IS and 0.09° internal rotation for FS. This indicates tibial components with IS being less resistant to torque over time as compared with tibial components with FS. With a sensitivity of 50% and specificity of 90%, y-axis rotation at a threshold of 0.7° is also predictive of aseptic loosening of the tibial component (Gudnason et al. 2017).

### Periprosthetic stress-shielding

Periprosthetic stress-shielding and bone loss is well known in the tibial metaphysis after TKA. Minoda et al. (2010) showed an approximate 40% relative BMD change medial and lateral to a tibial component cemented stem 2 years after surgery. Varus knees have been shown to have higher BMD in the more loaded medial tibial metaphysis prior to TKA intervention; however, knee alignment correction with TKA resulted in similar medial and lateral BMD values suggesting bone remodeling responding to load (Soininvaara et al. 2004). Heterogeneous BMD loss in relation to a central stem has been described up to 7 years’ follow-up (Hernandez-Vaquero et al. 2008). At 2 years’ follow-up we found periprosthetic bone loss of mean 10–14% of postoperative BMD values medial, lateral, anterior, and posterior to the IS. In comparison, periprosthetic bone changes around the FS was between mean –7% (anterior) and +1% to –2% medial, lateral and posterior to the stem. The reason was probably higher bone stress around the I-beam stem by torsional forces.

After 2 years the bone stress-shielding eased off in the IS group but increased in the FS group. We saw a correlation between MTPM at 5 years’ follow-up and the periprosthetic bone loss percentage under the medial as well as the lateral part of both types of tibial components at 2 years indicating a clinical significance of bone loss. Formerly only a relationship between the preoperative BMD in the knee region and postoperative cementless tibial component migration has been shown (Andersen et al. 2017).

### Polyethylene wear

We found a mean polyethylene insert wear-rate of 0.21 mm/year and 0.10 mm/year in tibial components with IS and FS, respectively. This wear may arise from articulate wear as well as backside wear on the metal-backed tibial components (Conditt et al. 2004, Collier et al. 2007). The locking mechanism (an anterior split) of the polyethylene insert as well as the average PE thickness was the same and could not explain the wear difference found between stem groups. Our method could not differentiate between polyethylene wear in the medial and lateral compartments but the magnitude was similar to other reports, though these generally showed a bit more wear in the medial compartment and a bit less in the lateral compartment (Conditt et al. 2004, Collier et al. 2007).

### Clinical outcomes

The AKS scores in healthy persons without TKA in the same age range as the patients in our study are a knee score of median 100 (70–100) and a function score of median 71 (17–100) (Bremner-Smith et al. 2004). At 5 years, our patients had a function score of mean 58 (49–67) with a marginal and statistically insignificant improvement of 5 points since baseline. This was far below the clinically relevant change of 35 points (Jacobs and Christensen 2009) but, in comparison with other TKA studies with 6–11 years’ follow-up, our patients scored only slightly lower (Ladermann et al. 2008) or comparably (Arthur et al. 2013, Breugem et al. 2014). The knee score of our patients increased by more than 50 points until 5 years to a mean 89 (84–94) points, which was only slightly lower than for healthy individuals. Knee flexion of 115° was similar between groups and similar to other reports (Faris et al. 2015).

### Limitations and strengths

Due to aging and morbidity, there was a marked dropout of 23 patients (of a total 54) at 5 years’ follow-up in this elderly patient group (mean age 77 years at time of inclusion). However, the dropouts were similarly distributed between groups (12 IS, 11 FS), and although it was higher than the anticipated sample size at 2 years, the study had sufficient power to detect group differences at 5 years. The strength of the study was that only the stem design differed between groups; the tibial components were otherwise alike and fixed with cement in the same manner and by the same surgeons.

In summary, at minimum 5 years’ follow-up there was less periprosthetic bone stress-shielding, superior fixation, less polyethylene wear, and similar clinical outcome on AKKS for tibial components with finned stem compared with tibial components with I-beam stem.

### Supplementary data

Tables 2, 4, and 5 are available as supplementary data in the online version of this article, http://dx.doi.org/10.1080/17453674.2019.1566510

## Supplementary Material

Supplemental Material
