# Supplementary material for: Superior fixation and less periprosthetic stress-shielding of tibial components with a finned stem versus an I-beam block stem: a randomized RSA and DXA study with minimum 5 years’ follow-up
Source: Acta Orthop. 2019 Jan 23;90(2):165–71. doi: 10.1080/17453674.2019.1566510 (PMC6461099; doi:10.1080/17453674.2019.1566510)
Supplement: Supplemental Material [file IORT_A_1566510_SM2090.pdf]

Supplementary data

Table 2. Measurement error for 29 double-examination stereoradiographs (15 IS and 14 FS)

	Translations (mm)				Rotations (°)			
Axis:	x	y	z	MTPM [a]	x	y	z	TR [b]
$Mean_{dif}$ [c]	0.01	0.00	0.03	0.03	0.07	0.06	0.01	0.05
SD_{dif} [d]	0.08	0.04	0.15	0.20	0.30	0.46	0.11	0.26
CR [e]	0.15	0.07	0.29	0.39	0.58	0.90	0.22	0.51
Minimum	–0.29	–0.09	–0.49	-0.30	–1.24	–1.32	–0.21	–0.29
Maximum	0.12	0.08	0.28	0.75	0.45	1.40	0.30	1.11

There was no statistically significant difference between IS and FS.
[a] MTPM: Maximum total point motion is an absolute migration parameter (migration vector).
[b] TR: Total rotation was calculated using the 3D Pythagorean Theorem (TR = square root ($x^2+y^2+z^2$).
[c] $Mean_{dif}$: Systematic error between two RSA double examinations (should optimally be 0).
[d] SD_{dif}: The standard deviation of the differences (SDdif) represents the random variation in the applied RSA method (precision).
[e] CR: The coefficient of repeatability (1.96 x SDdif) reflects the clinical precision on an individual basis.

Table 4. Bone mineral density change in the 3 ROIs below the tibial component presented as mean (95% CI) percentage change from the baseline values at 1, 2, and minimum 5 years

ROI	I-beam stem	Finned stem	p-value [a]
**Anterior/posterior view**			
ROI 1 (lateral to stem):			
1 year	–11 (–16 to –6)	–2 (–11 to 6)	0.2
2 years	–13 (–17 to –10)	–7 (–14 to 0)	0.2
5 years	–17 (–24 to –9)	–15 (–27 to –3)	0.8
ROI 2 (medial to stem):			
1 year	–13 (–16 to 10)	1 (–6 to 8)	0.001
2 years	–13 (–16 to –9)	–2 (–9 to 5)	0.02
5 years	–14 (–20 to –8)	–9 (–16 to –3)	0.3
ROI 3 (below stem):			
1 year	–6 (–8 to –3)	1 (–3 to 6)	0.01
2 years	–6 (–8 to –3)	–1 (–7 to 4)	0.3
5 years	–6 (–10 to –2)	–4 (–11 to 3)	0.9
Mean of anterior/posterior ROIs:			
1 year	–10 (–13 to –7)	0 (–6 to 6)	0.005
2 years	–11 (–13 to –8)	–3 (–10 to 2)	0.1
5 years	–12 (–18 to –7)	–9 (–17 to –1)	0.9
**Lateral view**			
ROI 1 (anterior to stem):			
1 year	–11 (–16 to –6)	–2 (–11 to –1)	0.2
2 years	–14 (–17 to –10)	–7 (–14 to 0)	0.2
5 years	–17 (–24 to –9)	–15 (–27 to –3)	0.8
ROI 2 (posterior to stem):			
1 year	–13 (–16 to –10)	1 (–6 to 8)	0.001
2 years	–13 (–16 to –9)	–2 (–9 to 5)	0.02
5 years	–14 (–20 to –8)	–9 (–16 to –3)	0.3
ROI 3 (distal to stem):			
1 year	–6 (–8 to –3)	1 (–3 to 6)	0.01
2 years	–6 (–8 to –3)	–1 (–7 to 4)	0.3
5 years	–6 (–11 to –2)	–3 (–11 to 3)	0.9
Mean of lateral ROIs:			
1 year	–10 (–13 to –7)	0 (–6 to 6)	0.005
2 years	–10 (–13 to –8)	–4 (–10 to 2)	0.1
5 years	–12 (–18 to –6)	–9 (–17 to –1)	0.9

[a] Two-sample Wilcoxon rank-sum (Mann–Whitney) test.
